# Complicated variation of simple renal cyst usually means malignancy: results from a cohort study

**DOI:** 10.1186/1477-7819-12-316

**Published:** 2014-10-15

**Authors:** XiaoJian Qin, Lin Ye, HaiLiang Zhang, Bo Dai, Yao Zhu, GuoHai Shi, DingWei Ye

**Affiliations:** Department of Urology, Fudan University Shanghai Cancer Center, Shanghai, China; Department of Oncology, Shanghai Medical College, Fudan University, Shanghai, China

**Keywords:** diagnosis, kidney neoplasm, renal cell carcinoma, simple renal cyst, treatment

## Abstract

**Background:**

Currently, simple renal cysts (SRCs) are not considered to warrant follow-up or specific treatment unless a patient presents symptomatically. By demonstrating malignant transformation of SRCs, we urge regular follow-up and timely surgical treatment in affected patients.

**Methods:**

From September 2002 to September 2010, we treated 31 cases of renal cell carcinoma derived from SRCs. Among these patients, in 14 cases a SRC was radiographically detected by computed tomography (CT) or magnetic resonance imaging (MRI) prior to operation, and malignant tumors were detected by pathological analysis following laparoscopic cyst decortication; 13 of these patients received supplementary radical nephrectomy within 2 months, whereas one patient chose to receive active surveillance. The other 17 patients exhibited SRCs and were monitored by ultrasound for over 6 months; surgical treatment was chosen if a complicated variation of SRCs was found during surveillance, detected by ultrasound, and confirmed by CT or MRI. Median follow-up was 60 months (30 to 126 months). All data analyzed were collected with informed consent, and the study was approved by the ethical committee of our institute.

**Results:**

Pathological studies confirmed early-stage clear-cell renal cell carcinoma in all of the cases, with Fuhrman grade I to III. In decortication-detected malignancies, supplementary radical nephrectomy exhibited residual tumor in 7 out of 13 cases; the patient who chose active surveillance remains free of recurrence for 78 months, and all other patients survived without disease at the last follow-up.

**Conclusions:**

Renal cell carcinoma may be detected incidentally in SRCs, and more attention should be paid to complicated variations of SRCs during surveillance, owing to the extremely high probability of malignancy. This interesting but alarming phenomenon might urge regular follow-up and timely surgical treatment in affected patients.

## Background

Simple renal cysts (SRCs) or Bosniak categorized class I renal cysts are the most common benign renal lesion, representing more than 70% of all asymptomatic renal masses. They can be found in over 50% of patients older than 50 years [1]. Currently, SRCs are not considered to warrant follow-up or specific treatment unless a patient presents symptomatically [2], because transformation of SRCs into renal cell carcinoma is extremely rare. Currently in urologic practices, patients with SRCs who are candidates for surgical treatment commonly receive cyst decortication, because this procedure offers a long-term cure, and is also an effective method of determining whether suspicious kidney cysts are benign. However, malignancies occasionally occur in presumed SRCs [3, 4], and our practice has observed that some SRCs have progressed into complex renal cysts, which were further confirmed by pathological analysis to be renal cell carcinoma. In this study, we present the first large cohort of patients with renal cell carcinoma derived from SRCs; our findings may challenge current management strategies for SRCs.

## Methods

From September 2002 to September 2010, a total of 31 patients with renal cell carcinoma derived from SRCs were admitted and treated in our department. Medical records were retrospectively collected and analyzed. The patients ranged from 24 to 68 years of age (median 52 years); 15 of them were male. On admission, the diameter of the renal cysts detected radiologically ranged from 1.5 cm to 10 cm (median 3.8 cm), with a history of renal cysts of between 4 days and 6 years (median 21 months).

Among these patients, 14 were assumed to have SRCs according to computed tomography (CT) or magnetic resonance imaging (MRI) prior to surgery, and malignant tumors were detected by pathological analysis after laparoscopic cyst decortications. In eight of these cases, frozen-section analyses were employed during the initial surgical procedure, with negative findings. After malignancy was confirmed by paraffin pathology, 13 of the patients were referred to undergo supplementary radical nephrectomy in 2 months (7 days to 50 days); one patient refused this procedure and received active surveillance.

Another 17 patients exhibited a history of SRCs for over 6 months (6 months to 6 years, median 36 months), and chose surgical treatment when, during surveillance, complicated variation of SRCs was identified. These cysts had first been detected by ultrasound examination, and CT or MRI, leading to the diagnosis of SRCs, further confirmed eight of these cases. These patients underwent ultrasound examination as surveillance every 6 to 12 months. After a complicated variation was screened out by ultrasound, CT or MRI was performed prior to surgery. Radical nephrectomy was conducted in nine of the patients, and the other eight received nephron-sparing surgery.

Sharply defined cysts displaying well-transmitted sound waves and an absence of echoes on ultrasonography are defined as SRCs, and complicated variations in ultrasound examination were defined as solid components, wall thickening, or internal echoes. In CT or MRI, complicated variations of renal cysts were termed complex renal cysts, or generally categorized as Bosniak class III or IV renal cysts, defined as cystic masses with thickened irregular or smooth walls or septa, in which measurable enhancement is present, or clearly malignant cystic masses that exhibit all criteria of category III, but also contain enhancing soft-tissue components adjacent to, but independent of, the wall or septum [2].

Median follow-up was 60 months (30 to 126 months). All data analyzed were collected with informed consent. Our study was approved by the ethical committee of Fudan University Shanghai Cancer Center.

## Results and discussion

All 14 cases of laparoscopic cyst decortication-detected malignancy were classified as grade I clear-cell renal cell carcinoma. Pathological analysis of paraffin embedded tissue samples following supplementary radical nephrectomy exhibited residual tumor in 7 cases out of 13; the patient who chose active surveillance has remained recurrence-free for 78 months. In the 17 SRC cases with complicated variations, all were diagnosed as clear-cell carcinoma by pathological studies, with Fuhrman grade I to III. The only case of grade III cancer was found in a 24-year-old woman, who had a Bosniak class IV renal cyst; 14 months later, a SRC was confirmed by CT and diagnosed as a clear-cell renal cell carcinoma with cystic change; the other 16 cases were diagnosed as cystic renal cell carcinoma or multilocular cystic renal cell carcinoma, of which 12 were grade I renal cell carcinoma. All of the patients have survived, and were without recurrence or metastases at the last follow-up.

The pathogenesis of SRCs remains unknown, and transformation of SRCs into renal cell carcinoma is extremely rare. A previous long-term report indicated that 2 out 61 SRC cases progressed to malignancy after 10 years of follow-up [5]; the SRCs continued to increase in size over 10 years, and occasionally increased rapidly, particularly in younger patients. The size seemed not to be an important factor for progression of SRCs to renal cell carcinoma. No apparent, specific pattern has been observed in neoplasm-bearing renal cysts [5]. Currently, patients are referred to surgical intervention based only upon symptoms, and clinicians potentially pay less attention to their occurrence, which in turn causes patients to neglect seeking further care. Not every patient with an SRC should be referred for surgery; however, the potential malignancy of SRCs should be taken into consideration.

A limited number of renal cell carcinoma cases are detected incidentally as SRCs, and unfortunately, they are radiographically identical to true SRCs. Currently, there are no specific preoperative diagnostic tools that allow safe identification. Pathological examination and immunohistochemistry analysis is always required to detect the presence of malignant cells in the cyst wall. Cases of renal cell carcinoma detected incidentally as SRCs generally exhibit a thin wall with no solid portion upon gross and microscopic examination on frozen sections. Our study and others [6] found that intra-operative frozen-section analyses do not lead to a correct diagnosis in many cases, most probably because of the low nuclear grade that makes it difficult to distinguish malignant cells from normal cells. In most cases, the final pathological examination exhibits a thin layer of cuboidal clear cells that cover part of the cyst.

With regard to possible malignancies, interventions to renal cysts, such as puncture and aspiration, should be employed with caution; even when biopsies of the cyst wall are obtained, malignant cells could potentially be missed, owing to the heterogeneous nature of the malignant transformation. In this study, cancer cells did not cover the cyst wall universally in specimens after laparoscopic decortication, and supplementary radical nephrectomy only detected residual tumor in about half of the cases. However, since it is possible that the malignant transformation of the cyst wall occurred in a limited manner, and decortication alone also exhibits the possibility of a cure, as in one of our patients, there was still no evidence of disease 78 months after decortication. Thus, if further aggressive surgical procedures were not preferred, active surveillance would also be acceptable in these patients; however, supplementary radical nephrectomy was generally recommended for patients in whom renal cell carcinoma was detected in cyst decortication specimens. Patients who do not choose supplementary radical nephrectomy should be informed that nearly half these cases exhibit residual tumor that is a potential source of recurrence.

The complicated variation of SRCs during surveillance is another situation of concern. It is commonly understood that complicated variation of SRCs is principally derived from hemorrhagic cysts, usually as a result of trauma, enlargement, or bleeding diathesis. As hemorrhagic cysts resolve, they develop a residual calcification centrally or within the cyst wall that becomes thickened and develops septa; the cyst then becomes multilocular or multilobular, essentially acquiring the features of a complex cyst [7–9]. To date, there is an insufficient amount of data to discuss the histopathologic patterns of cysts with complicated variation; moreover, a limited number of SRC cases with complicated variation were reported, and all were confirmed to be malignant [10–12].

**Figure 1:**
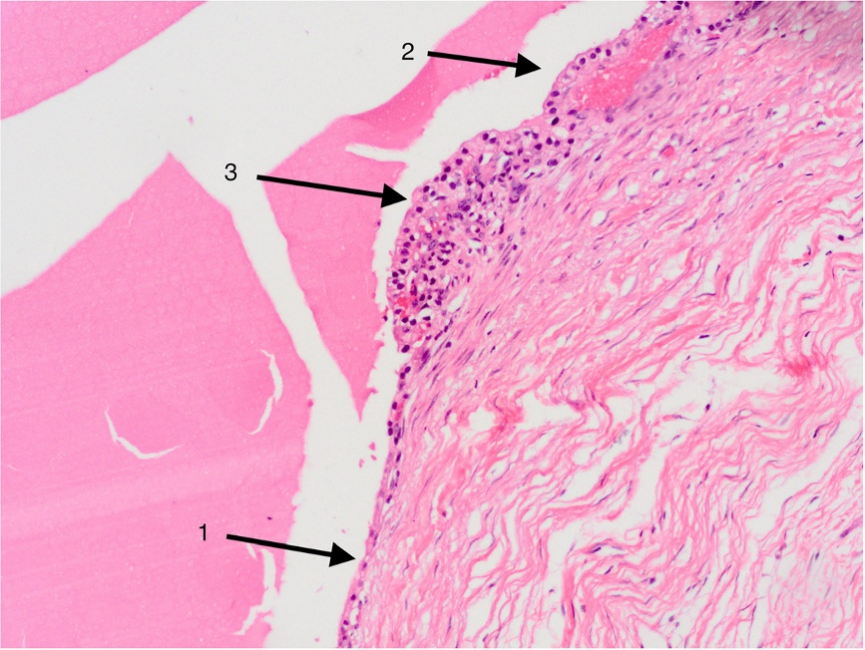
**Malignant transformation of renal cyst (×200; H & E stain).** Normal (arrow 1) and neoplastic (arrows 2, 3) cells lining macroscopic cysts, indicating that malignant complex renal cysts might be a progression of malignant simple renal cysts that transform from benign tissue.

It is known that malignancies in Bosniak III or IV renal cysts are frequent, as approximately 40 to 60% of class III and 85 to 100% of class IV cysts are malignant [7–9]; however, in our study, all 17 cases with a history of SRCs followed by complicated variation during surveillance turned out to be malignant, without exception, and the probability was markedly higher than reported in the general population. To explain the extremely high proportion of malignancy, it is reasonable to speculate that malignant complex renal cysts are progressions of malignant SRCs and that growth is facilitated because malignant SRCs form a thickened wall; hemorrhage may develop into a multilocular cyst or nodules in a cyst. In cystic renal cell carcinoma or multilocular cystic renal cell carcinoma, the cysts are usually lined by a single layer of epithelial cells or lack an epithelial lining. The lining cells may be flat or plump, and their cytoplasm ranges from clear to pale. Occasionally, the lining consists of several layers of cells, or a few small papillae may be present, as described previously [13] and found in some of our cases (Figure 1). This potentially serves as part of the evidence that malignant complexes renal cysts are progressions of malignant SRCs; however, further investigation regarding the origin of malignant complex renal cysts is warranted.

The prognosis appears to be good in SRCs with malignant transformations, particularly in patients whose malignancy was incidentally found in SRCs [14]; to date, there is no consensus on the stratification criteria on the pathological stage of these carcinomas. Considering that the prognosis of this kind of malignant lesion seems to be far better than common renal cell carcinoma with solid lesions, we do not think the TNM (tumor-node-metastasis) system, where the T stage is based on the size of the tumor, is suitable for these cystic lesions. As to the present cases, pathological stages would better be termed ‘localized’ or ‘early stage’ , because there is no evidence of local invasions or distant metastases; however, more malignant tumors are also possible. We reported grade III renal cell carcinoma with cystic changes, and a previous study has reported Bellini ductal carcinoma with metastases that was fatal [5].

We have presented the largest cohort of SRCs with malignant transformations to date, and discussed the strategies utilized to manage SRCs. Our investigation has its limitations. Some cases of SRCs were diagnosed only by ultrasound analysis, whereas some presumed cases of SRCs might not be real SRCs because ultrasonography alone could miss some small lesions of complicated variation; however, this indicates that greater attention should be paid to the diagnosis of SRCs. It is generally believed that ultrasonography plays a limited role in the evaluation of cystic renal masses and should be reserved for characterization of simple or minimally complex renal cysts (containing one or two hairline-thin septa). Ultrasonography alone should not be utilized to differentiate surgical from nonsurgical complex cystic renal masses [2]. Radiological (CT and MRI) findings were similar in the majority of cystic renal masses. In some cases, MRI depicted additional septa, thickening of the wall or septa, or enhancement, which might lead to an upgraded Bosniak cyst classification that might affect case management [15]. Usually, ultrasound is chosen for the surveillance of patients with SRCs. As a rule, sharply defined cysts with well-transmitted sound waves and an absence of echoes on ultrasonography are defined as SRCs, whereas CT or MRI should further evaluate any complexity deviating from this. Our results present experience in dealing with such a rare but troublesome situation, and this study may alert our colleagues to investigate this in their own patients.

## Conclusions

Renal cell carcinoma may be detected incidentally in SRCs. Frozen-section analyses were of limited use in distinguishing malignant from benign cysts; however, in certain cases, supplementary radical nephrectomy or active surveillance might be acceptable if renal cell carcinoma is identified after cyst decortication. Regular follow-up is warranted in SRCs, and more attention should be paid to complicated variation of SRCs during surveillance, owing to the extremely high probability of malignancy. Ultrasound examination is highly recommended for SRCs every 6 months, and CT or MRI are also suggested, if possible. Upon confirmation of complicated variation, patients should be referred to surgery immediately; both radical nephrectomy and nephron-sparing surgery are acceptable. Prognosis is good in malignant transformations of SRCs following surgery.

## Abbreviations

CT: computed tomography
H & E: hematoxylin and eosin
MRI: magnetic resonance
SRC: simple renal cyst
TNM: tumor-node-metastasis.
